# Supplementary figures and images for: A novel HIV triple broadly neutralizing antibody (bNAb) combination-based passive immunization of infant rhesus macaques achieves durable protective plasma neutralization levels and mediates anti-viral effector functions
Source: PLoS One. 2024 Nov 11;19(11):e0312411. doi: 10.1371/journal.pone.0312411 (PMC11554116; doi:10.1371/journal.pone.0312411)

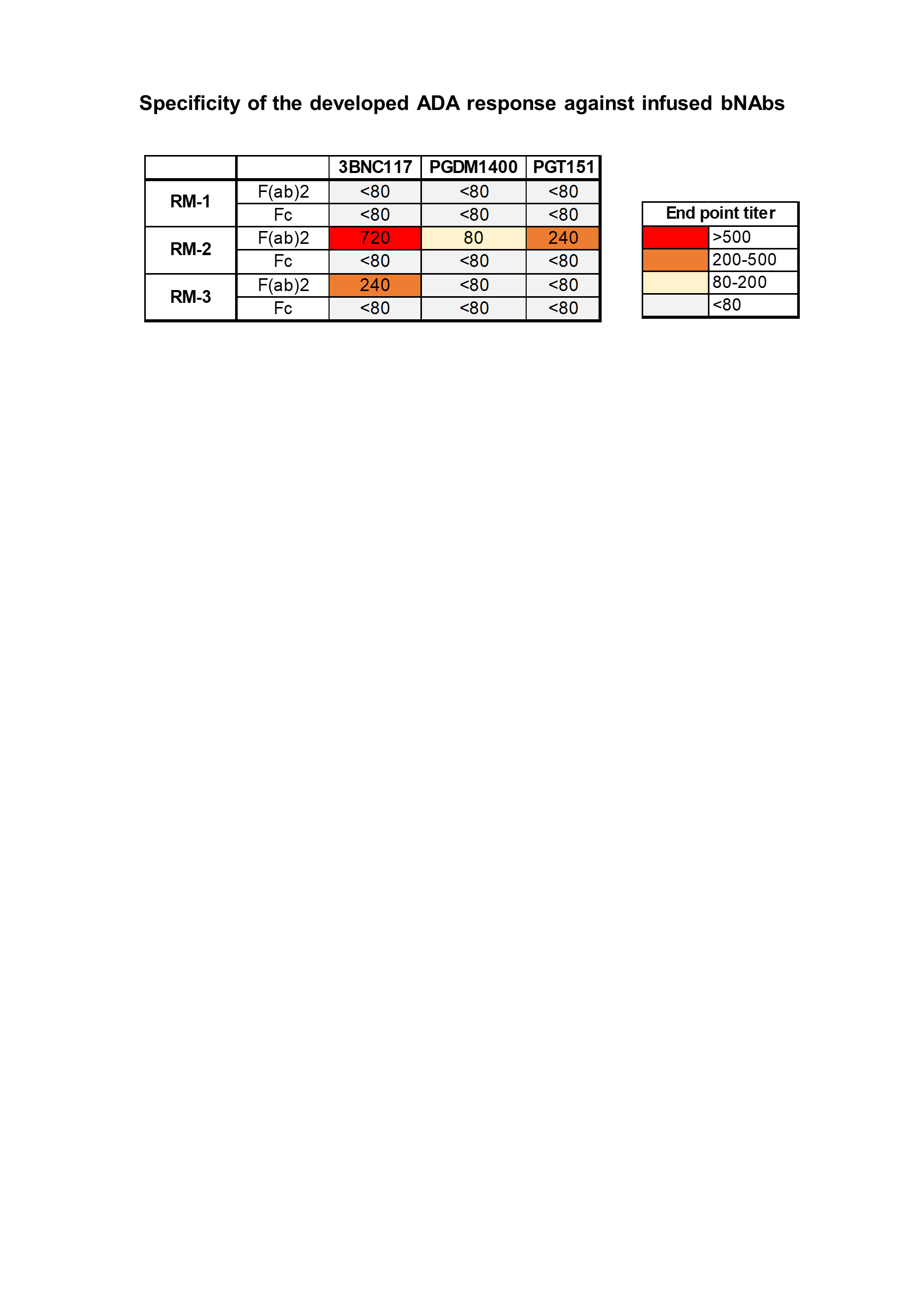

Supplement: S1 Fig — (TIF) [file pone.0312411.s001.TIF]

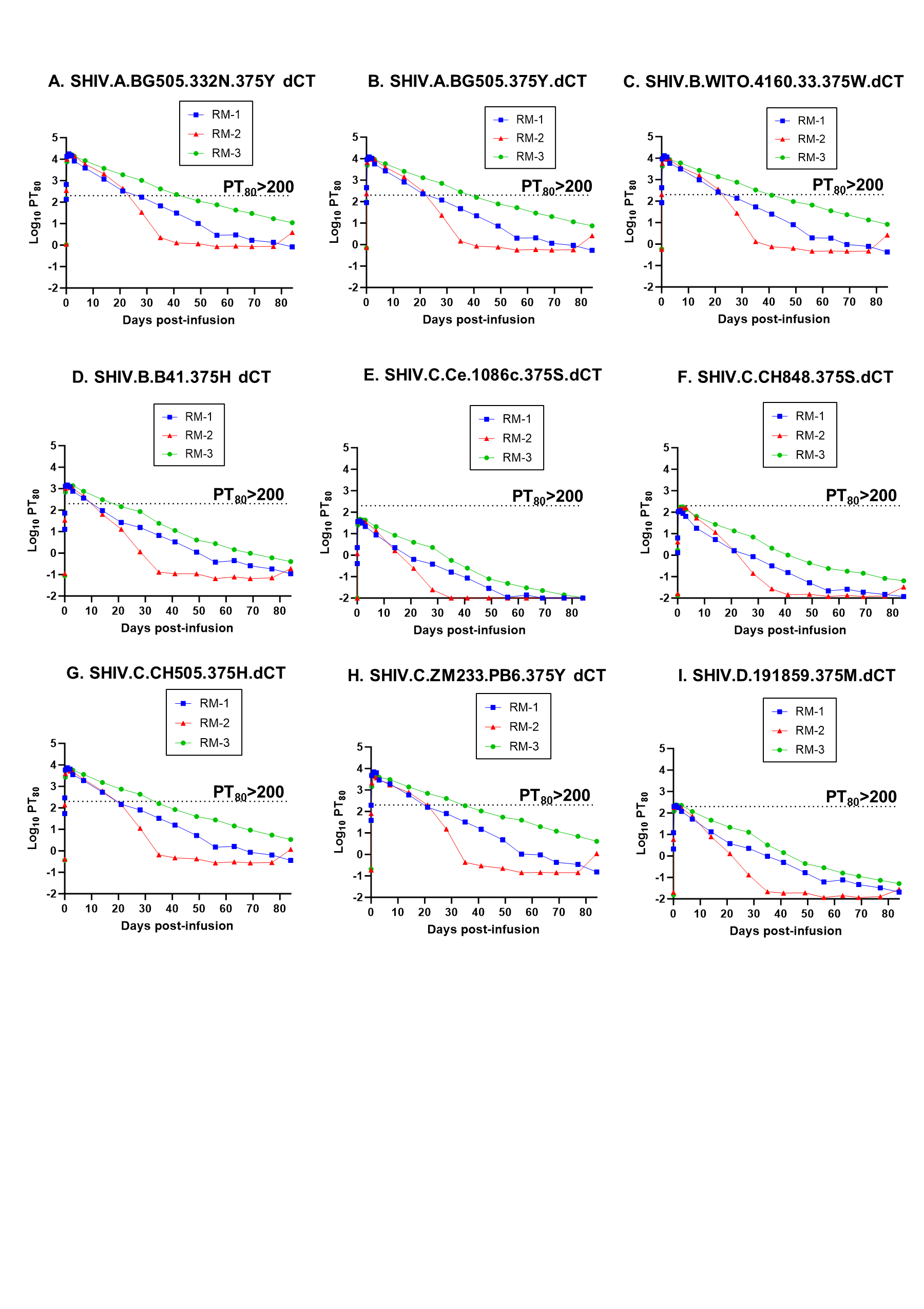

Supplement: S2 Fig — PT80 values of Rh bNAb combination against (A) SHIV.A.BG505.332N.375Y.dCT (B) SHIV.A.BG505.375Y.dCT (C) SHIV.B.WITO.4160.33.375W.dCT (D) SHIV.B.B41.375H.dCT (E) SHIV.C.Ce.1086c.375S.dCT (F) SHIV.C.CH848.375S.dCT (G) SHIV.C.CH505.375H.dCT (H) SHIV.C.ZM233.PB6.375Y.dCT and (I) SHIV.D.191859.375M.dCT. Dashed line represents PT80>200of the antibody combination against the SHIV variants in rhesus macaques. (TIF) [file pone.0312411.s002.TIF]
