# Supplementary material for: A novel HIV triple broadly neutralizing antibody (bNAb) combination-based passive immunization of infant rhesus macaques achieves durable protective plasma neutralization levels and mediates anti-viral effector functions
Source: PLoS One. 2024 Nov 11;19(11):e0312411. doi: 10.1371/journal.pone.0312411 (PMC11554116; doi:10.1371/journal.pone.0312411)
Supplement: Attachment — Submitted filename: Response_to_Reviewers_RG.docx [file pone.0312411.s003.docx]

Journal Requirements:

When submitting your revision, we need you to address these additional requirements.

1. Please ensure that your manuscript meets PLOS ONE's style requirements, including those for file naming. The PLOS ONE style templates can be found at

[https://journals.plos.org/plosone/s/file?id=wjVg/PLOSOne_formatting_sample_main_body.pdf](https://urldefense.com/v3/__https:/journals.plos.org/plosone/s/file?id=wjVg*PLOSOne_formatting_sample_main_body.pdf__;Lw!!Aer6R9v1Nk4!7jcDo6bvt3VI2U1bUwMlC3WI4IKtDfY3ZMaONA3Awhj2wH3Up5ynlnxMxxYwBxU34MJ40dBHlP63pJegRAw1gLWV$) and

[https://journals.plos.org/plosone/s/file?id=ba62/PLOSOne_formatting_sample_title_authors_affiliations.pdf](https://urldefense.com/v3/__https:/journals.plos.org/plosone/s/file?id=ba62*PLOSOne_formatting_sample_title_authors_affiliations.pdf__;Lw!!Aer6R9v1Nk4!7jcDo6bvt3VI2U1bUwMlC3WI4IKtDfY3ZMaONA3Awhj2wH3Up5ynlnxMxxYwBxU34MJ40dBHlP63pJegRBD-iFHk$)

**Response:** Thank you for this suggestion. We have now formatted our manuscript based on PLOS One’s style requirement and named the files as suggested.

2. Thank you for stating the following in the Acknowledgments Section of your manuscript:

"This work was supported by NIH P01 5P01AI131276 (S.R.P, G.G.F, A.C, G.S, R.R.A. C.C), NIH 5UM1AI164566 (A.C, S.R.P, G.G.F), NIH 5P01AI117915 (S.R.P). and NIH’s Office of the Director, Office of Research Infrastructure Programs, P51OD011132. The ADCC assay was performed at Duke Human Vaccine Institute (Durham, NC) as part of the Primate AIDS Vaccine Evaluation Group (PAVEG) contract HSN272201800004C. The rhesusization, expression, and purification of antibodies were partially funded by NIH P40 OD028116 (D.M.M., K.E.). The funders had no role in study design, data collection, and interpretation, or the decision to submit the work for publication."

We note that you have provided additional information within the Acknowledgements Section that is not currently declared in your Funding Statement. Please note that funding information should not appear in the Acknowledgments section or other areas of your manuscript. We will only publish funding information present in the Funding Statement section of the online submission form.

Please remove any funding-related text from the manuscript and let us know how you would like to update your Funding Statement. Currently, your Funding Statement reads as follows:

"This work was supported by NIH P01 5P01AI131276 (S.R.P, G.G.F, A.C, G.S, R.R.A. C.C), NIH 5UM1AI164566 (A.C, S.R.P, G.G.F), NIH 5P01AI117915 (S.R.P). and NIH’s Office of the Director, Office of Research Infrastructure Programs, P51OD011132. The ADCC assay was performed at Duke Human Vaccine Institute (Durham, NC) as part of the Primate AIDS Vaccine Evaluation Group (PAVEG) contract HSN272201800004C. The rhesusization, expression, and purification of antibodies were partially funded by NIH P40 OD028116 (D.M.M., K.E.).

"Please include your amended statements within your cover letter; we will change the online submission form on your behalf.

**Response:** Thank you for this suggestion. We have now removed any funding-related information from the manuscript's text and the Acknowledgement section. The online funding statement will be updated to the following.

"This work was supported by NIH P01 5P01AI131276 (S.R.P, G.G.F, A.C, G.S, R.R.A. C.C), NIH 5UM1AI164566 (A.C, S.R.P, G.G.F), NIH 5P01AI117915 (S.R.P). and NIH’s Office of the Director, Office of Research Infrastructure Programs, P51OD011132. The ADCC assay was performed at Duke Human Vaccine Institute (Durham, NC) as part of the Primate AIDS Vaccine Evaluation Group (PAVEG) contract HSN272201800004C. The rhesusization, expression, and purification of antibodies were partially funded by NIH P40 OD028116 (D.M.M., K.E.). The funders had no role in study design, data collection and analysis, decision to publish, or preparation of the manuscript."

We have also included this updated version in the cover letter.

3. Thank you for stating the following financial disclosure:

"This work was supported by NIH P01 5P01AI131276 (S.R.P, G.G.F, A.C, G.S, R.R.A. C.C), NIH 5UM1AI164566 (A.C, S.R.P, G.G.F), NIH 5P01AI117915 (S.R.P). and NIH’s Office of the Director, Office of Research Infrastructure Programs, P51OD011132. The ADCC assay was performed at Duke Human Vaccine Institute (Durham, NC) as part of the Primate AIDS Vaccine Evaluation Group (PAVEG) contract HSN272201800004C. The rhesusization, expression, and purification of antibodies were partially funded by NIH P40 OD028116 (D.M.M., K.E.). "

Please state what role the funders took in the study.  If the funders had no role, please state: "The funders had no role in study design, data collection and analysis, decision to publish, or preparation of the manuscript."

If this statement is not correct you must amend it as needed.

Please include this amended Role of Funder statement in your cover letter; we will change the online submission form on your behalf.

**Response:** Thank you for this suggestion. We have updated the financial disclosure as suggested (find below) and have included it in the cover letter.

"This work was supported by NIH P01 5P01AI131276 (S.R.P, G.G.F, A.C, G.S, R.R.A. C.C), NIH 5UM1AI164566 (A.C, S.R.P, G.G.F), NIH 5P01AI117915 (S.R.P). and NIH’s Office of the Director, Office of Research Infrastructure Programs, P51OD011132. The ADCC assay was performed at Duke Human Vaccine Institute (Durham, NC) as part of the Primate AIDS Vaccine Evaluation Group (PAVEG) contract HSN272201800004C. The rhesusization, expression, and purification of antibodies were partially funded by NIH P40 OD028116 (D.M.M., K.E.). The funders had no role in study design, data collection and analysis, decision to publish, or preparation of the manuscript."

4. Thank you for stating the following in the Competing Interests section:

"S.R.P serves as a consultant for Merck, Moderna, Pfizer, Hoopika, Dynavax, and GSK on their CMV vaccine programs, and has led sponsored programs with Merck and Moderna on CMV vaccines. Other authors have no conflict of interest to disclose."

Please confirm that this does not alter your adherence to all PLOS ONE policies on sharing data and materials, by including the following statement: "This does not alter our adherence to  PLOS ONE policies on sharing data and materials.” (as detailed online in our guide for authors [http://journals.plos.org/plosone/s/competing-interests).](https://urldefense.com/v3/__http:/journals.plos.org/plosone/s/competing-interests).__;!!Aer6R9v1Nk4!7jcDo6bvt3VI2U1bUwMlC3WI4IKtDfY3ZMaONA3Awhj2wH3Up5ynlnxMxxYwBxU34MJ40dBHlP63pJegRBlNP0F-$)  If there are restrictions on sharing of data and/or materials, please state these. Please note that we cannot proceed with consideration of your article until this information has been declared.

Please include your updated Competing Interests statement in your cover letter; we will change the online submission form on your behalf.

**Response:** Thanks for bringing this up. We have now updated our Competing interest section to the following.

“S.R.P serves as a consultant for Merck, Moderna, Pfizer, Hoopika, Dynavax, and GSK on their CMV vaccine programs, and has led sponsored programs with Merck and Moderna on CMV vaccines. Other authors have no conflict of interest to disclose. This does not alter our adherence to PLOS ONE policies on sharing data and materials.”

We have also included this in our cover letter and in the manuscript text in lines 673-676.

5. When completing the data availability statement of the submission form, you indicated that you will make your data available on acceptance. We strongly recommend all authors decide on a data sharing plan before acceptance, as the process can be lengthy and hold up publication timelines. Please note that, though access restrictions are acceptable now, your entire data will need to be made freely accessible if your manuscript is accepted for publication. This policy applies to all data except where public deposition would breach compliance with the protocol approved by your research ethics board. If you are unable to adhere to our open data policy, please kindly revise your statement to explain your reasoning and we will seek the editor's input on an exemption. Please be assured that, once you have provided your new statement, the assessment of your exemption will not hold up the peer review process.

**Response:** Thank you for the suggestion. All data has been uploaded on Github, accessible from github.com/Riagoswami1/Triple_bNAb_PK_infant_NHP. Data access will be made public, upon provisional acceptance of the manuscript. This information has now been updated in the manuscript text in line 649-650.

Additional Editor Comments:

Please revise figure 2: the ADA induction is graphed in a way that is not reflecting the actual data. ADA presence is only determined experimentally to appear on day 35 while the graph indicates a linear rise from day 3 to day 35, which is likely not correct.

**Response:** We thank the Editor for this suggestion. We have now changed Figure 2 per Editor’s suggestion. Figure 2A-C now indicate the levels of the 3 antibodies in plasma and Figure 2D-F demonstrate ADA levels against the 3 antibodies. We have also made the necessary changes in the figure legend (lines 187-192).


[Note: HTML markup is below. Please do not edit.]

Reviewers' comments:

Reviewer's Responses to Questions

**Comments to the Author**

1. Is the manuscript technically sound, and do the data support the conclusions?

The manuscript must describe a technically sound piece of scientific research with data that supports the conclusions. Experiments must have been conducted rigorously, with appropriate controls, replication, and sample sizes. The conclusions must be drawn appropriately based on the data presented.

Reviewer #1: No

Reviewer #2: Partly

2. Has the statistical analysis been performed appropriately and rigorously?

Reviewer #1: Yes

Reviewer #2: Yes

3. Have the authors made all data underlying the findings in their manuscript fully available?

The [PLOS Data policy](https://urldefense.com/v3/__http:/www.plosone.org/static/policies.action*sharing__;Iw!!Aer6R9v1Nk4!7jcDo6bvt3VI2U1bUwMlC3WI4IKtDfY3ZMaONA3Awhj2wH3Up5ynlnxMxxYwBxU34MJ40dBHlP63pJegRER3X_4w$) requires authors to make all data underlying the findings described in their manuscript fully available without restriction, with rare exception (please refer to the Data Availability Statement in the manuscript PDF file). The data should be provided as part of the manuscript or its supporting information, or deposited to a public repository. For example, in addition to summary statistics, the data points behind means, medians and variance measures should be available. If there are restrictions on publicly sharing data—e.g. participant privacy or use of data from a third party—those must be specified.

Reviewer #1: No

Reviewer #2: Yes

4. Is the manuscript presented in an intelligible fashion and written in standard English?

PLOS ONE does not copyedit accepted manuscripts, so the language in submitted articles must be clear, correct, and unambiguous. Any typographical or grammatical errors should be corrected at revision, so please note any specific errors here.

Reviewer #1: Yes

Reviewer #2: Yes

5. Review Comments to the Author

Please use the space provided to explain your answers to the questions above. You may also include additional comments for the author, including concerns about dual publication, research ethics, or publication ethics. (Please upload your review as an attachment if it exceeds 20,000 characters)

Reviewer #1: Dankwa et al. assessed the safety, pharmacokinetics, ex vivo neutralizing capacity, and functionality of a trivalent rhesuzised HIV bNAb combination that was passively transferred to infant rhesus monkeys. Available data suggest that the treatment is well tolerated, however, the study consists of only three animals. The half-life of bNAbs was relatively short, and was substantially shortened in one animal, coinciding with the generation of ADA. Analysis of serum samples showed intact binding, neutralizing, ADCC and ADCP function of the transferred bNAb cocktail. bNAbs were also detected in mucosal samples, which may be relevant for protection from vertical transmission. Preventive efficacy of the treatment against a panel of HIV variants was calculated using the PK-data-derived PT80 biomarker, which correlated with the experimentally derived ID80 titers.

The manuscript is well written and clear. The data provide insight into the PK and tissue penetration of bNAbs in the infant NHP model, however, it is not convincingly justified how the PT80 biomarker can give any indication of the PE to be expected in bNAb treated infants.

**Response.** We agree with the reviewer's concern that the PT_80_ biomarker has not been established to predict prevention from perinatal and breast milk transmission. We have modified the manuscript text to highlight that future studies to investigate whether a PT_80_ biomarker threshold of >200 against perinatally and breast milk-exposed HIV variants is sufficient to provide ≥90% preventative efficacy against vertical HIV transmission will be crucial (Line 441-447). We have removed the claim that in perinatal or breast milk transmission PT80>200 is equivalent to PE≥90%. We have modified the manuscript text and updated Figure 4B and 4D and associated legends to reflect the change.

Comments:
1. The rapid generation of ADA in 2 out of 3 NHP is a concern. Is this related to the young age of the animals and is this frequency also expected in human infants? It would be helpful if this aspect was discussed in greater detail.

**Response.** We thank the reviewers for these questions. We have now included a paragraph (Lines 390-409) to discuss the potential contribution of age, immunization regimen, dose, route and frequency of administration, and host-specificity on the development of ADA responses against infused bNAbs.

2. Please clarify in the manuscript how data in figure 4B were generated for the present experiment. Are these data based on average serum Ab concentrations across all three RMs, or only for one representative animal?

**Response.** We apologize for the lack of clarity in our manuscript methods. The data shown in Figure 4B are based on median PT_80_ values of the combination regimen for each SHIV variant. We have now clarified this in Figure 4B legend.

3. The authors predict an PE of over 90% based on the PT80 biomarker. This prediction is in my view incorrect since it assumes that serum PT80 similarly predicts efficacy against adult (sexual) transmission, as observed in AMP, and infant horizontal transmission. Either the authors need to convincingly justify why PT80 should similarly predict PE for both routes of transmission, or the assumption ‘that universal infant prophylaxis early-after birth, in areas of HIV seroprevalence could provide protection from perinatal and breastmilk HIV transmission’ should be removed, and, throughout the whole manuscript, the interpretation of the PT80 biomarker data should not extrapolate to efficacy in infants.

**Response.** We agree with the reviewer's concern that the PT_80_ biomarker has not been established to predict prevention from perinatal and breast milk transmission. We have modified the manuscript text to highlight that future studies to investigate whether a PT_80_ biomarker threshold of >200 against perinatally and breast milk-exposed HIV variants is sufficient to provide ≥90% preventative efficacy against vertical HIV transmission will be crucial (Line 441-447). We have removed the claim that in perinatal or breast milk transmission PT80>200 is equivalent to PE≥90%. We have modified the manuscript text and updated Figure 4B and 4D and associated legends to reflect the change.

Reviewer #2: Dankwa et al. describe an important study investigating the pharmacokinetics and potency of an optimized combination of three broadly neutralizing antibodies (bnAbs) in infant rhesus macaques. The study is well designed, experiments mostly technically sound, and the manuscript is well written. The manuscript can benefit from addressing the following points:

1. Fig. 1 should be labeled to specifiy that SHIV.C.CH505.375H.dCT was used for neutralization assay.

**Response.** We thank the reviewer for this suggestion. Figure 1A has now been re-labeled.

2. The authors should repeat all neutralization assay using full length, WT CH505 Env (Fig. 3B) in addition to the data they present for the variant with the cytoplasmic tail deleted.

**Response.** We thank the reviewer for this suggestion. In a previous manuscript from our group (PMID: 33177194), we demonstrated that the neutralization potency of the 3 infused bNAbs against WT CH505 infectious molecular clone was well correlated with that of the SHIV.CH505.375H.dCT variant (Figure 1 of PMID 33177194). Since SHIV.C.CH505.375H.dCT is a tier-2 clade C next-generation SHIV that can infect and replicate in infant RMs (PMID: 31488511) as well as recapitulate the viral replication dynamics and immunopathogenesis of HIV infection in humans (PMID: 31217249), in this manuscript we selected SHIV.CH505.375H.dCT. We are limited by the restricted plasma volume that was available from the monkeys at each time to be able to perform neutralization assays with both WT CH505 and SHIV.CH505.375H.dCT concurrently.

3. It would be helpful to provide more data on the ADCC assay, including raw data, and calculations.

**Response.** We thank the reviewer for this suggestion. We have now included more detail on calculations for the ADCC assay under the method section in lines 602-614 and have also uploaded the data in Github. Data will be publicly available from github.com/Riagoswami1/Triple_bNAb_PK_infant_NHP, upon manuscript acceptance.

4. It would be helpful to identify the epitope(s) of the anti-drug antibodies developed in the rhesuses macaques.

**Response.** We thank the reviewers for suggesting this experiment. We have performed additional experiments to assess the specificity of the developed ADA response against the 3 infused antibodies. The ADAs were found to be specific for the F(ab)2 region of each of the 3 antibodies. The data is now included as supplemental figure 1 and discussed in lines 209-211. The methods for performing the assay are described in lines 538-544 and 550-551. Previous studies have reported similar development of ADA response against the variable, but not constant region of passively infused bNAbs (PMID: 26444083, 30704961). Unfortunately, we could not further assess the specificity of the ADAs to heavy or light chains of the variable region due to the restricted volume of plasma that could be collected at each time point from these infant monkeys.

6. PLOS authors have the option to publish the peer review history of their article ([what does this mean?](https://urldefense.com/v3/__https:/journals.plos.org/plosone/s/editorial-and-peer-review-process*loc-peer-review-history__;Iw!!Aer6R9v1Nk4!7jcDo6bvt3VI2U1bUwMlC3WI4IKtDfY3ZMaONA3Awhj2wH3Up5ynlnxMxxYwBxU34MJ40dBHlP63pJegRHSC_W0d$)). If published, this will include your full peer review and any attached files.


If you choose “no”, your identity will remain anonymous but your review may still be made public.


**Do you want your identity to be public for this peer review?** For information about this choice, including consent withdrawal, please see our [Privacy Policy](https://urldefense.com/v3/__https:/www.plos.org/privacy-policy__;!!Aer6R9v1Nk4!7jcDo6bvt3VI2U1bUwMlC3WI4IKtDfY3ZMaONA3Awhj2wH3Up5ynlnxMxxYwBxU34MJ40dBHlP63pJegRCnNjJJ3$).

Reviewer #1: **Yes:** Frank Wegmann

Reviewer #2: No
